# *Viburnum lantanoides* (Adoxaceae): Leveraging Epicotyl Seed Dormancy Characterization to Optimize Seedling Regeneration Protocols

**DOI:** 10.3390/plants15131985

**Published:** 2026-06-26

**Authors:** Todd J. Rounsaville, Nora M. Bello, Molly E. Dieterich Mabin, Emily K. Johnson

**Affiliations:** 1 U.S. Department of Agriculture, Agricultural Research Service, U.S. National Arboretum, 10300 Baltimore Ave., Beltsville, MD 20705, USA; 2 U.S. Department of Agriculture, Agricultural Research Service, Northeast Area Office, 10300 Baltimore Ave., Beltsville, MD 20705, USA; nora.bello@usda.gov; 3 Oak Ridge Institute for Science and Education (ORISE), P.O. Box 117, Oak Ridge, TN 37831, USA

**Keywords:** biodiversity, conservation, germplasm, epicotyl dormancy, seedling, *Viburnum*

## Abstract

Efficient seed regeneration of woody plants is of practical importance for horticulture and conservation practitioners. Seeds of the genus *Viburnum* are known to be difficult to germinate, as mechanisms of morphophysiological dormancy breaking can vary significantly among species, necessitating unique treatments to promote embryo growth and overcome physiological dormancy barriers. In this study, our objectives were (1) to characterize the class of seed dormancy for *V. lantanoides*, and (2) to optimize a protocol for seed-based regeneration for the species. Specifically, we evaluated warm and cold stratification treatments of increasing length and assessed the effectiveness and timing of seed germination. Results indicate that *V. lantanoides* seeds exhibit deep simple epicotyl morphophysiological dormancy (MPD), for which a sequence of warm–cold–warm temperatures is required to complete germination processes. We further assessed probability and time to emergence for radicles, cotyledons, and true leaves, and found that the combination of 12-week warm stratification followed by 12-week cold stratification resulted in the fastest and most effective method for seedling regeneration. This study represents an initial exploratory report on seed dormancy and regeneration in *Viburnum* section *Pseudotinus* and is intended to enhance physiological understanding of *V. lantanoides* seeds in support of conservation and horticultural efforts.

## 1. Introduction

The ability to successfully and efficiently germinate seeds is of practical relevance to professionals across the agricultural industry. For wild species, and woody plants in particular, protocols for seed-based regeneration are often either non-existent or unsuitable, failing to produce consistent germination [1]. As a result, growers often resort to a brute-force approach—directly sowing seed into field rows and waiting, often for years, on ambient conditions to break dormancy and promote germination. Disadvantages of this approach include steady seed attrition via predators and seed degradation by soil microorganisms, as well as inefficient use of field space [2].

In many instances, seed availability may be limited, underscoring the need for efficient recovery of seed lots. Examples include conservation professionals working with rare and threatened wild species; gene bank staff and cooperators recovering preserved seeds from long-term storage; and plant breeders harvesting seed from hand-pollinations [3,4].

To be efficient with the regeneration of small and valuable seed lots, overcoming seed dormancy is a primary concern. Seeds are considered to be dormant when germination (emergence of radicle and/or shoot) is blocked under favorable conditions [5]. In dormant seeds, specific environmental cues including light, water, and temperature are required to alleviate one or more dormancy factors [6].

Accordingly, a diverse range of dormancy mechanisms have evolved in seed plants to maximize regeneration across environmental gradients [7]. At the highest level, Baskin and Baskin [8] classify dormancy into five hierarchical classes, which we describe briefly; (1) seeds with physical dormancy (PY) are impermeable to water and dormancy is overcome via opening of a ‘water gap’, generally via high-temperatures or physical scarification; (2) physiological dormancy (PD) results from a physiological inhibiting mechanism and is broken by warm and/or cold stratification; (3) morphological dormancy (MD) occurs in seeds with underdeveloped embryos for which suitable moisture, temperature, and light–dark requirements promote embryo growth and germination; (4) morphophysiological dormancy (MPD) is characterized by the presence of both PD and MD barriers; and (5) combinational dormancy is characterized by the presence of both PY and PD barriers [8,9].

Knowledge of a seed’s dormancy type and specific cues for dormancy breaking is therefore critical to facilitate the production of genetically diverse plant materials required for preservation/conservation efforts and horticultural improvement. Furthermore, documentation of juvenile plant regeneration (e.g., seeds and seedlings) has increasingly been recognized as a vital, yet neglected, resource for biodiversity monitoring, niche modeling, and policymaking [10,11,12].

The genus *Viburnum* (Adoxaceae) includes ca. 165 species of woody plants from temperate and subtropical systems throughout the Northern Hemisphere as well as mountainous regions of South America and southeastern Asia [13]. Viburnum is a popular ornamental crop commonly used in cultivated landscapes and subjected to breeding, hybridization, and selection efforts for over 80 years [3,14]. Yet, seeds of viburnum are regarded as particularly difficult and slow to germinate [15,16,17]. A number of seed dormancy barriers have been recognized within the genus [9], though many have been overcome by tailored dormancy-breaking treatments [18,19,20]. However, for the majority of viburnum taxa there are no published reports of seed regeneration protocols [1,21]. The importance of propagation efforts is further underscored by the ongoing spread of Viburnum Leaf Beetle for the past three decades [22] and its negative impact on U.S. viburnum taxa. This pest causes defoliation, resulting in reduced plant fecundity and increased death of susceptible taxa [23].

**Figure 1:**
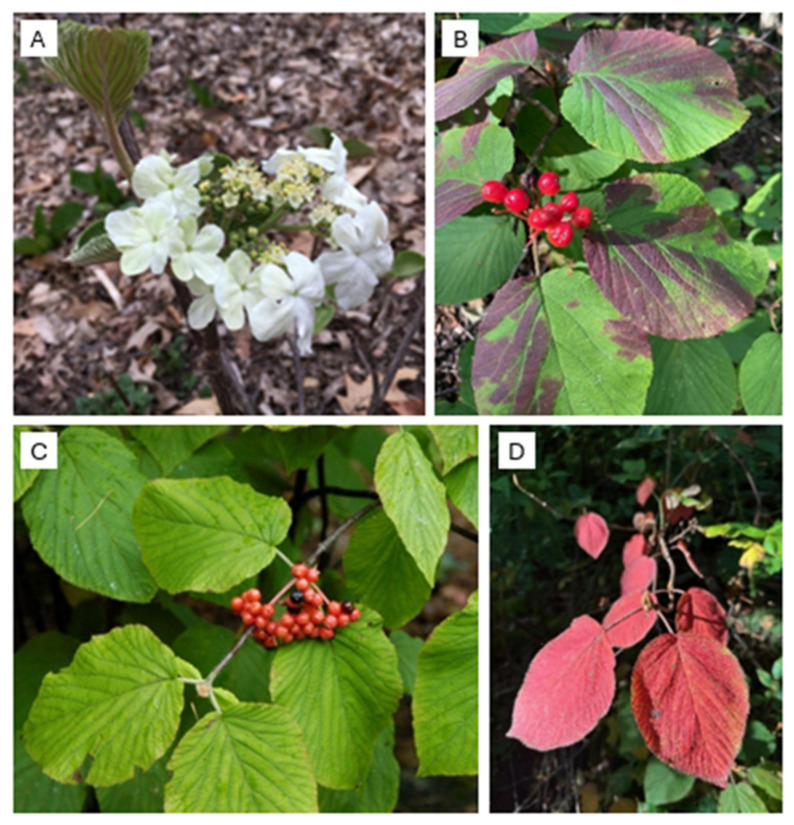
*Viburnum lantanoides*: (**A**) lacecap inflorescence showing sterile marginal flowers (Massachusetts); (**B**) maturing fruits with varicolor late-summer foliage (New Hampshire); (**C**) early maturing (purple–back) drupes (New York); (**D**) mid-autumn fall color (West Virginia). Image credits: Michael S. Dosmann (**A**), Rick J. Lewandowski (**C**).

Of special interest for this study is *Viburnum lantanoides* Michx., colloquially known as ‘hobblebush’. *V. lantanoides* is a common shrub within mixed mesophytic forests of New England and the Canadian Maritime Provinces and is found patchily at higher elevation sites in the southern Appalachians [24]. Ornamentally, *V. lantanoides* is noteworthy for being among the first woody plants to flower in the spring, and one of only nine of 165 viburnum species to have showy, sterile marginal flowers (Figure 1A) [25]. Bright and varicolored fall foliage presented on horizontally oriented stems at the time of fruit set contributes to its value as a multi-season ornamental shrub (Figure 1B–D). Although C.S. Sargent [26] noted that hobblebush was “one of the most beautiful plants of our [U.S.] flora”, he went on to note it was also “the most difficult of all our native shrubs to cultivate”. In comparison to other U.S. viburnums, hobblebush has a greater affinity for shade and moist conditions, frequently forming thickets in the forest understory [27]. Previous reports have noted challenges with seed propagation of the species [16,25,28].

For many viburnum taxa studied, seeds are reported to exhibit some form of MPD [29]. Twelve levels of MPD have been described based on the temperature regimes (i.e., warm and/or cold stratification) and the sequence of temperatures required to promote growth of the embryo, radicle, and epicotyl [8,17]. *Viburnum* section *Pseudotinus* contains four species including *V. lantanoides* and there are no previous reports of MPD level within the clade. Based on published accounts among *Viburnum* spp., MPD level does not appear to be conserved within phylogenetic clade, continental endemism, or biome type [20,29,30]. Taxon-specific characterization of MPD level is therefore an important step in understanding recruitment dynamics and optimizing protocols for plant regeneration.

The objectives of this study were (1) to characterize the class of seed dormancy types for *V. lantanoides* and thus provide an initial report informative of the *Pseudotinus* clade, and (2) to optimize a protocol for seed-based *V. lantanoides* regeneration. This study aims to support seed ecologists and researchers of evolution and diversification in *Viburnum.* Insight will further improve regeneration procedures for plant breeders, gene banks, and conservation/restoration practitioners.

## 2. Results

### 2.1. Seed Physiology and Dormancy Classification

**Figure 2:**
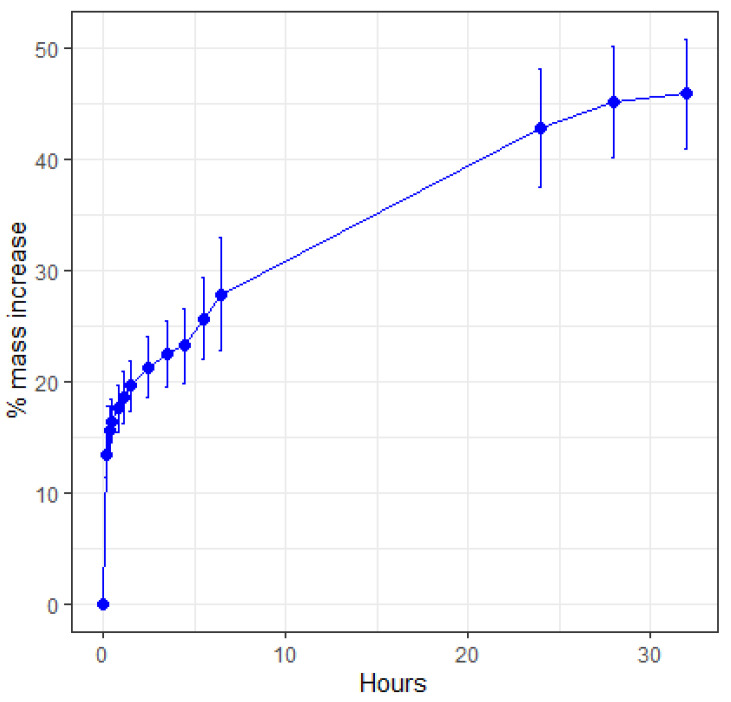
Seed permeability: Observed seed mass during imbibition time course, expressed as a percentage relative to baseline. Each datapoint represents the average measurement from 10 seeds. Whiskers indicate SD.

*V. lantanoides* seeds were found to be permeable to water, with a rapid increase in relative mass of approximately 28% within the first 8 h of imbibition, and over 40% within 24 h (Figure 2).

**Figure 3:**
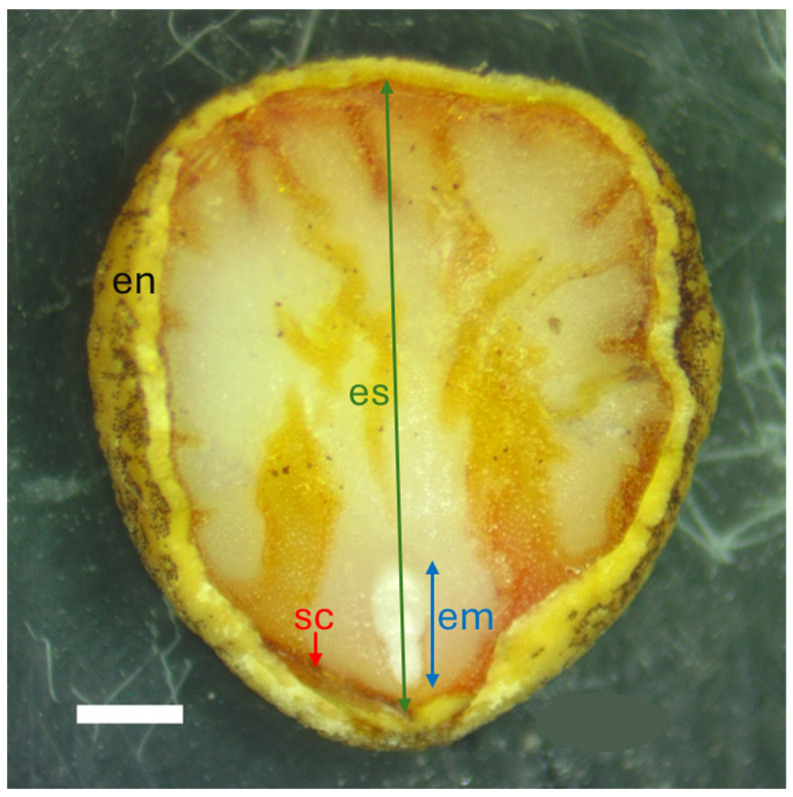
Illustration of seed in longisection prior to initiation of temperature cycling regimes (i.e., time 0). Shown are embryo (em), endosperm (es), seed coat (sc), and endocarp (en). The ratio of the length of em (blue arrow) over the length of es (green arrow) was used to define the E:S ratio. Scale bar = 1 mm.

**Figure 4:**
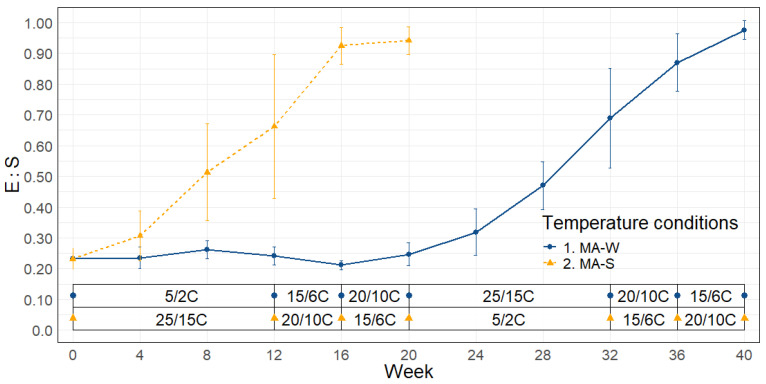
Observed embryo to seed ratio (E:S) throughout the temperature cycling treatments MA-W and MA-S, as presented along the *x*-axis. Each datapoint represents the average measurement from 10 seeds. Whiskers indicate SD.

**Figure 5:**
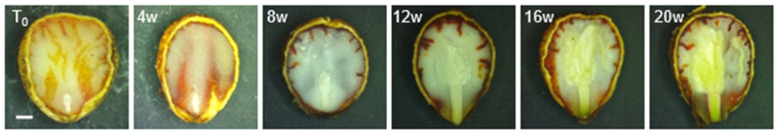
Illustration of embryo growth over time under the temperature cycling treatment MA-S, characteristic of conditions received in situ. Seeds were initiated at summer temperatures (25/15 °C) for twelve weeks, followed by early-autumn temperatures (20/10 °C) for weeks 12 to 16, and finally late-autumn temperatures (15/6 °C) for weeks 16 to 20. Scale bar = 1 mm.

Dissection of fresh, mature seeds used for embryo development assessment revealed underdeveloped, spatulate embryos (Figure 3). Embryos were 1.30 ± 0.18 mm in length (mean ± SD) representing an E:S ratio of 0.232 ± 0.034 prior to initiation of treatments. Under MA-W, initial incubation of seeds at 5/2 °C simulating winter temperatures failed to promote embryo growth for approximately the first 20 weeks of incubation (Figure 4); during this time E:S remained below 0.25 in all cases. Meanwhile, by 12 weeks of exposure to temperatures of 25/15 °C, embryos showed E:S of 0.69 ± 0.16 and 0.66 ± 0.23 under treatments MA-W and MA-S, respectively (Figure 4 and Figure 5).

**Figure 6:**
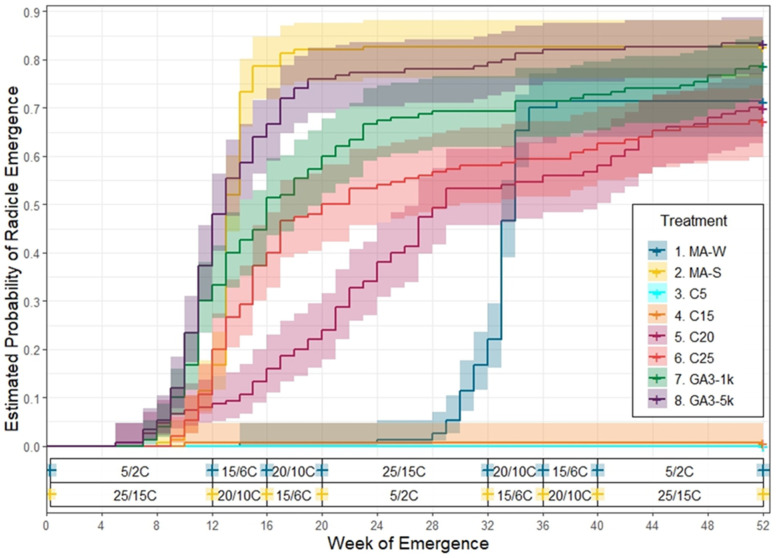
Seed dormancy characterization study: Estimated cumulative probability of radicle emergence throughout a 52-week observation period. Move along treatment (MA-W and MA-S) temperatures are presented along *x*-axis. Remaining treatments maintained constant day/night temperatures of 5/2 °C (C5), 15/6 °C (C15), 20/10 °C (C20), and 25/15 °C (C25, GA3-1k, and GA3-5k). Bands indicate 95% confidence limits.

Overall, radicle emergence was observed to initiate after exposure to 25/15 °C for approximately eight weeks; this corresponds to approximately week 28 of treatment MA-W or week 8 of treatment MA-S (Figure 6). Furthermore, for seeds under both MA-W and MA-S, we observed a sharp increase in the occurrence of radicle emergence during the two-week period following their movement from 25/15 °C to 20/10 °C (weeks 32–34 and 12–14, respectively). For both MA-W and MA-S treatments, this two-week interval accounted for approximately 60% and 69% of seeds showing radicle emergence, respectively. After 52 weeks of incubation, the proportion of seeds showing radicle emergence ranged from 68 to 84% across treatments, except for C5 and C15, for which radicle emergence was observed in 0% and less than 1% of seeds, respectively.

Hormonal seed priming with both 1000 and 5000 ppm GA3 successfully promoted radicle emergence at 25/15 °C. Control seeds (C25) reached 50% radicle emergence after 20 weeks. At this time, GA3-1k and GA3-5k treatments exhibited radicle emergence of 60% and 76% of seeds, respectively, though formal testing was not conducted due to design limitations.

Shoot growth, defined as the emergence of cotyledons and epicotyls, was observed for approximately 88% of MA-S seedlings by week 52 of the study (data not presented). Meanwhile, between 75 and 96% of seedlings subjected to MA-W, C20, C25, GA3-1k, and GA3-5K treatments were alive by week 52 but failed to exhibit any signs of cotyledon emergence or shoot growth. Treatments C5 and C15 were not assessed for shoot growth due to a lack of radicle emergence at these constant temperatures.

### 2.2. Seedling Regeneration Protocol: Radicle Emergence

**Figure 7:**
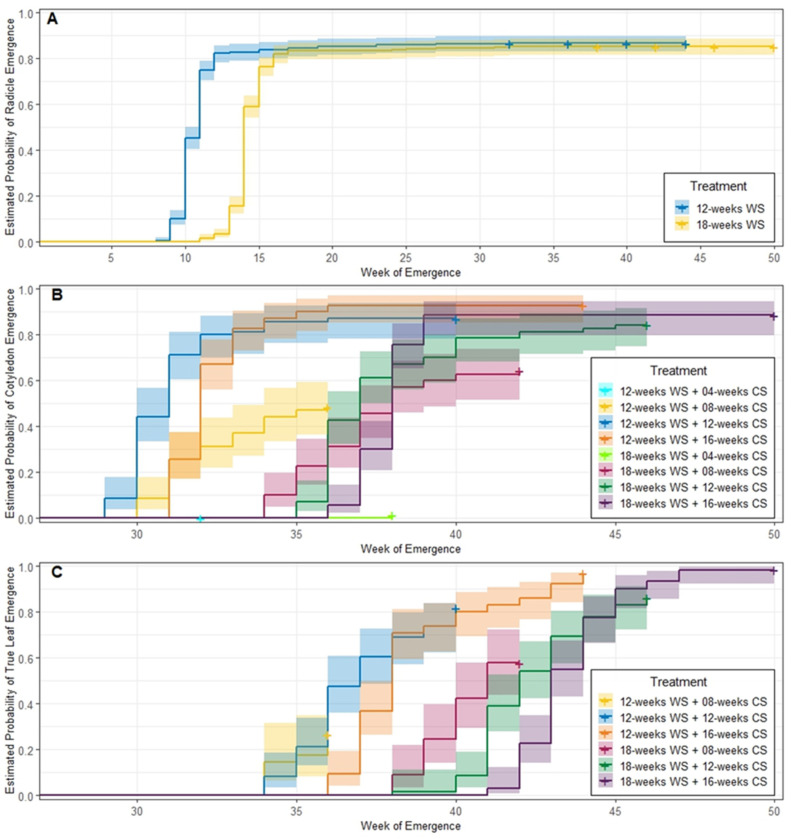
Optimization of seedling regeneration protocols: Estimated cumulative probabilities of (**A**) radicle emergence, (**B**) cotyledon emergence, and (**C**) true leaf emergence throughout the observation period. Treatments consisted of combinations of warm stratification [(WS): 12 vs. 18-week-long] and cold stratification [(CS): 4, 8, 12 and 16-week-long]. Bands indicate 95% confidence limits.

Overall, the probability of radicle emergence was high across treatments (mean estimates of 82.2%, 95%CI: [77.8, 86.0]% and 83.5% [79.1, 87.1]% for 12- and 18-week-long warm stratification (WS) treatments, respectively; *p* = 0.64), with a combined censoring (i.e., non-emerged radicles) of 14% among the 800 seeds used in this study. Meanwhile, the length of WS affected (*p* < 0.0001) the timing of radicle emergence, such that emergence of radicles was accelerated in response to the 12-week treatment compared to the 18-week treatment. Specifically, the hazard ratio for radicle emergence between treatments was estimated at 2.4 (95% CI: [1.9, 3.1]). That is, the hazard, or instantaneous rate of radicle emergence, was over twice as large for seeds subjected to 12- vs. 18-week WS and peaked approximately at weeks 10–11 and 14 for each treatment, respectively (Figure 7A). Recall that WS treatments differed in the duration of the first of two stages, leading to a total WS duration of 12 or 18 weeks. The first stage of the 12- and 18-week-long WS consisted of 6 or 12 weeks, respectively, at 25/15 °C, followed by a second stage at 20/10 °C for 6 weeks. The duration of the second stage was identical for both WS treatments.

### 2.3. Seedling Regeneration Protocol: Cotyledon Emergence

For seeds subjected to 4-week-long cold stratification (CS), the probability of successful cotyledon emergence was found to be close to zero, and estimated at 1.2% [0.2, 5.9]% regardless of duration of WS (interaction *p*-value = 0.25). For this reason, seeds subjected to 4-week-long CS were excluded from further analyses on the timing of subsequent phenological stages (i.e., cotyledon and first leaves emergence). For longer CS treatments combined with either 12- or 18-week-long WS, the probability of successful cotyledon emergence increased (*p* < 0.0001) to an estimated 56.5% [48.1, 64.6]% following 8 weeks of CS, and to 85.3% [78.4, 90.2]% and 90.4% [84.1, 94.3]% following 12 and 16 weeks of CS, respectively.

Meanwhile, the timing of cotyledon emergence, and therefore of shoot dormancy breaking, was found to be dependent on the combination of WS and CS treatments (Figure 7B). Specifically, CS for 12 or 16 weeks was more effective than 8-week CS at speeding cotyledon emergence if preceded by a 12-week-long WS period (estimated hazard ratios of 2.6 [1.6, 4.2] and 2.5 [1.6, 4.0], *p*-values = 0.0015 and 0.0028, respectively). However, when CS was preceded by an 18-week-long WS, there was no evidence for differences among 8, 12, or 16 weeks of CS in timing to cotyledon emergence (*p* > 0.99 in all cases).

### 2.4. Seedling Regeneration Protocol: Leaf Emergence

For leaf emergence, the probability of success was driven by duration of CS (*p* < 0.001), regardless of how long seeds had been exposed to WS. In particular, the probability of successful leaf emergence was estimated at 41.2% [27.8, 56.2]% following 8 weeks of CS and increased to 84.3% [74.4, 90.9]% (*p* = 0.003) and then to 97.8% [90.6, 99.5]% (*p* < 0.05) when CS was extended to 12 and 16 weeks of duration, respectively.

In addition, our data showed evidence of a combined effect of WS and CS duration on timing to leaf emergence (*p* = 0.066). Overall, the 12-week-long WS sped up first leaf emergence compared to the 18-week-long WS, regardless of CS length (*p* < 0.005 in all cases). However, combination of 12-week-long WS followed by 12-week-long CS resulted in the fastest timing to successful first leaf emergence, as indicated by hazard ratios estimated at 2.1 [1.4, 3.2] relative to 12-week WS and 16-week CS, and at 4.7 [2.1, 10.9], 8.5 [5.4, 13.6] and 4.5 [3.1, 6.6] relative to 18-week WS and CS ranging from 8 to 12 to 16 weeks, respectively. That is, the speeding effect of 12 weeks of WS on timing to first leaves emergence was maximized if combined with 12 weeks of CS (Figure 7C).

## 3. Discussion

The objectives of this study were to facilitate an initial characterization of seed dormancy in *Viburnum lantanoides* and to promote optimization of seedling regeneration. To this end, we evaluated different temperatures and the sequence/duration of these temperatures on seed and seedling physiological responses. Our work suggested that experimental assays commonly used to characterize seed dormancy can be further leveraged to streamline protocols for seedling regeneration. More specifically, our results suggested that a sequence of warm–cold–warm stratification seems to be required to complete the germination process in *V. lantanoides.* While admittedly preliminary in nature, our work further provided guidance on selecting optimal stratification lengths in support of the unique need for controlled regeneration activities. In addition, these data provided context for *V. lantanoides* recruitment dynamics in situ and offered additional insight for conservation efforts.

### 3.1. Seed Physiology and Dormancy Classification

Although Gill and Pogge [31] observed that some *Viburnum* species possess impermeable seed coats, we found that *V. lantanoides* seeds with endocarps rapidly imbibed water. Baskin and Baskin [32] note that for dry seeds, an increase in mass of at least 20% after 24 h of imbibition is indicative of seed permeability. We observed seed mass increases above 40% within 24 h of imbibition, indicating that *V. lantanoides* is unlikely to possess a physical dormancy (PY) component and therefore scarification treatments are likely unnecessary to promote germination [33].

Consistent with previous reports in viburnum, the embryos of *V. lantanoides* were underdeveloped at seed maturity [16], indicating a morphological dormancy (MD) component in the species [9]. The E:S ratio of 0.232 ± 0.034 observed among mature *V. lantanoides* seeds is within the range of previously reported congeners, including 0.104 and 0.102 for *V. betulifolium* and *V. parvifolium*, respectively [18], 0.204 for *V. lantana* [19], 0.28 ± 0.18 for *V. plicatum* var. *formosanum* [20], and 0.14 for *V. tinus* [34]. Giersbach [29] noted two U.S. taxa, *V. nudum* and *V. scabrellum*, displayed only MD by virtue of both radicles and shoots emerging within a few weeks of WS. Given our findings that WS alone is insufficient to promote shoot growth, we speculate that a physiological dormancy (PD) barrier is likely to also be present in *V. lantanoides*; we thus turn our consideration to morphophysiological dormancy (MPD).

Twelve subclasses of MPD have been recognized, and these are nested within two main categories, namely simple and complex, for which embryos develop in response to WS vs. CS, respectively [8]. Our observations that embryo growth (Figure 4) and radicle emergence (Figure 6) occur during WS is consistent with *V. lantanoides* seeds falling within the simple category of MPD. To our knowledge, most viburnums reported to have MPD are also ‘simple’ [15,17,29], with the exception of *V. plicatum* var. *formosanum,* for which embryo growth and radicle emergence were optimized at 5 °C, an indication of complex MPD [20].

We also consider the factors required to promote shoot emergence (i.e., growth of epicotyls) in relation to radicle emergence. Our observations that shoot growth only occurred among the MA-S treatment would suggest that (1) the CS period was likely obligatory to break shoot dormancy, and (2) CS may only be effective at breaking shoot dormancy after WS had promoted radicle emergence. We therefore speculate that the seed of *V. lantanoides* likely possesses deep simple epicotyl MPD, commonly referred to as simply “epicotyl dormancy” [9]. Epicotyl dormancy also occurs in seeds with PD, where dormancy of roots and shoots is sequentially broken by the accumulation of thermal time (e.g., CS duration) such that shoot dormancy breaking requires a longer stratification period than for roots [8]. A delayed emergence of shoots (relative to roots) is thought to offer a competitive advantage, such that seedlings with predeveloped root systems enter the growing season developmentally ahead of non-germinated seeds [17].

Deep simple epicotyl MPD has previously been reported for the majority of studied U.S. viburnums including *V. acerifolium*, *V. dentatum*, *V. lentago*, *V. prunifolium*, and *V. rufidulum* [29]. This commonality among most U.S. taxa may be the result of a convergent adaptation to a shared cool-temperate biome [30]. Nevertheless, there is evidence to suggest that different MPD subclasses or alternative dormancy types may be found in viburnum species endemic to warmer environments in the southern U.S., such as *V. australe*, *V. nitidum*, and *V. obovatum* [35]. To our knowledge, seed dormancy for these species has not been characterized. For example, although Giersbach [29] concluded that *V. scabrellum* seeds possess only MD, this taxon is now recognized as a synonym and southern variant of *V. dentatum*, for which Giersbach [29] did observe epicotyl dormancy. In general, MPD is more common in colder biomes with comparatively higher, more consistent precipitation, as opposed to warmer and drier climates where MPD is found less frequently [36]. *Viburnum* spp. endemic to warmer climates of Asia and Europe have been reported to show little (i.e., non-deep) or no epicotyl dormancy [34,37].

The sole New World member of section *Pseudotinus*, *V. lantanoides,* is thought to have entered North America in the mid-Miocene through the Bering Land Bridge and have arisen from an immediate ancestor that was already cold-adapted [30]. Notably, there are no published reports of dormancy among Old World Pseudotinus taxa, represented by *V. furcatum* (Japan and Korea), *V. nervosum* (southern China), and *V. sympodiale* (central China to Taiwan). Future studies are warranted to explore seed dormancy adaptations of this clade across a broader array of environmental conditions.

Seed dormancy is considered an evolved trait for delaying germination until environmental conditions are most favorable for seedling growth and survival [8]. Data presented herein suggest that for *V. lantanoides*, seeds are likely to mature in late summer at temperatures of approximately 25/15 °C. Following dispersal, ambient temperatures would then be expected to signal embryo growth, followed by radicle emergence and rooting ahead of extended winters in cool-temperate forests. This adaptation would presumably enable seeds to be stabilized in the soil during winter, during which exposure to cold temperatures presumably breaks shoot dormancy and allows for cotyledon emergence and epicotyl growth at the first spring. In support of this proposed mechanism, another northern U.S. species with deep simple epicotyl MPD, *Viburnum acerifolium,* was found to complete germination during the second spring, approximately 15 months post-dispersal [38]. In that study, comparatively later seed maturation (late autumn) curtailed radicle emergence until autumn of the following year, suggesting that summer temperatures may be necessary to initiate embryo growth [38].

Fruits of *V. lantanoides* mature asynchronously, changing color from red to dark purple (Figure 1C) from ca. early August to late September [25]. It is possible that this phenology acts as a bed-hedging strategy for seeds from the same individual, where early dispersed seeds receive sufficient WS to produce radicles prior to the first winter, whereas delayed fruit maturation would suspend radicle emergence to the following summer, as in *V. acerifolium* [38]. Temporal variation in fruit maturity can be an optimal maternal strategy as it increases the heterogeneity of dispersal agents and environmental conditions at the time of seed predation [39,40]. Accordingly, asynchronous maturity of fruits from the same maternal plant can lead to endogenous changes in dormancy sensitivity among MPD seeds as well as the prevailing stratification conditions at the time of dispersal [41,42]. In situ seed ecology studies would be necessary to fully elucidate these dynamics in *V. lantanoides*.

### 3.2. Seedling Regeneration

Leveraging our qualitative findings from the move-along study proved useful in screening conditions for further in-depth testing to optimize seedling regeneration protocols. We first noted that CS was ineffective at initiating embryo development and radicle emergence. Conversely, a two-stage WS provided by summer (12 weeks at 25/15 °C) and early-autumn (4 weeks at 20/10 °C) temperatures led to >95% of total radicle emergence within the MA-S treatment. We chose to further explore the two-stage WS period by considering two potential durations of summer temperatures (i.e., 6 vs. 12 weeks; first stage of WS), each followed by 6 weeks at early-autumn temperatures (second stage of WS). The total duration of WS was therefore 12 and 18 weeks long to include the entire two-stage conditioning period.

For many *Viburnum* spp. with deep simple epicotyl MPD, previous reporting noted 20/10 °C as the optimal day/night temperature for radicle emergence [17]. This is consistent with our results herein, where we found above 80% estimated probability of radicles emerged during the early-autumn (20/10 °C) phase of warm stratification following summer conditioning (i.e., either 6 or 12 weeks at 25/15 °C). Our data indicate that the shorter WS treatment consisting of 6-week summer + 6-week autumn led to faster radicle emergence, compared to longer WS (i.e., 12-week summer + 6-week autumn). By comparison, Barton [15] found that WS requirements for radicle emergence in viburnum ranged from 8 to 12 weeks (*V. opulus*) up to 24–68 weeks (*V. acerifolium* and *V. dentatum*) at 30/20 °C.

After radicle emergence, we expected CS to be a requirement for breaking shoot dormancy, thereby promoting cotyledon emergence and epicotyl development. We found that the shortest CS treatment consisting of only 4 weeks proved ineffective for shoot growth under either WS treatment. In *Helleborus thibetanus* seeds with deep simple epicotyl MPD, at least seven weeks of CS were required to break shoot dormancy, though CS duration requirements decreased for seedlings with longer radicles [43]. This effect seems consistent among seeds with epicotyl dormancy, whereby roots seem to require elongation before shoots can become sensitive to the effects of CS [44]. In her studies of epicotyl dormancy in multiple genera, Barton [15] noted that approximately 2–16 weeks of CS were required to break shoot dormancy. Barton [15] reported on seeds from five viburnum species whose CS requirements to overcome shoot dormancy included both extremes, ranging from 2 to 4 weeks (*V. dentatum*) to 12–16 weeks (*V. dilatatum*).

For *V. lantanoides*, our findings that extended CS seem obligatory for seed regeneration could limit the species’ sexual recruitment to regions with comparatively warmer climates, or within their extant range during years with milder winters. Presently, the southernmost extant populations of *V. lantanoides* are restricted to high elevation sites near the southern terminus of the Appalachian range [24]. In recent years, this species has been noted as extirpated from Georgia, formerly its southernmost station [35]. It is also possible that more southern stations of the species are adapted with reduced CS requirements, as seems to be true for the *V. dentatum* complex [29].

Taken together, data from this exploratory study provide preliminary practical guidance to help inform seed-based regeneration of *V. lantanoides*. Specifically, we found that the time to emergence of both cotyledons and true leaves was optimized by temperature cycling consistent with warm and cold stratification, starting with summer (6 weeks at 25/15 °C), followed by autumn (6 weeks at 20/10 °C) and finally winter (12 weeks at 5/2 °C) temperature (Figure 7). This 12-week WS → 12-week CS protocol reflected an approximately 25% reduction in duration relative to documented regeneration protocols that recommend a 21-week WS → 11-week CS to yield approximately 43% total germination [16]. In the present study, the emergence probability of cotyledons and true leaves under the 12-week WS → 12-week CS treatments were 86.6% [76.5, 92.8]% and 81.9% [66.8, 91.3]%, respectively.

### 3.3. Practical Applications

In many instances, the most rapid and effective regeneration protocols may be desirable, particularly for reducing intergenerational time in plant breeding applications [3]. Protocols enabling fast, high-percentage regeneration could be especially advantageous in developing solutions for integrating Viburnum Leaf Beetle (VLB) resistance in *V. lantanoides* [22]. Varying levels of susceptibility to VLB have been found in both New and Old World taxa, suggesting available pools of germplasm for resistance breeding efforts [22,45], and *V. lantanoides* is currently considered to be moderately susceptible [46].

Plant breeders may also seek to utilize *V. lantanoides* for its ornamental qualities, particularly its colorful fall foliage and ‘lacecap’ type inflorescences (Figure 1), one of only two U.S. species with this flower trait. Although there are over 250 recognized viburnum cultivars [47], to our knowledge, there are no commercially available *V. lantanoides* selections in the nursery trade. Annual U.S. sales of deciduous and evergreen viburnum each exceed $38 M, a combined $76 M crop for domestic wholesale and retail [48]. In the U.S., a relatively small number of exotic *Viburnum* species and interspecific hybrids dominate commercial sales. However, demand for so-called ‘native’ plants has increased in recent years, providing an opportunity to introduce previously underutilized species to U.S. markets [49,50].

It is worth noting that although longer stratification treatments delayed time-to-emergence events, our data provided no evidence for any reduction in emergence output for any of the phenological stages considered. For instance, we found that the longest treatment combination consisting of WS (18 weeks) plus CS (16 weeks) led to estimated probabilities of cotyledon and true leaf emergence of 88.0% [78.1, 93.8] and 98.3% [83.8, 99.8], respectively (Figure 7). Longer stratification treatments may be a desirable option in selected applications, including commercial growers who may rely on minimally heated high-tunnels or greenhouses to produce seedlings for horticulture and restoration plantings. In these cases, longer stratification periods may be more cost effective without any apparent detriment to seed regeneration. Nevertheless, an important consideration for sowing under outdoor/ambient conditions is that seeds should be sown with ample time for WS prior to winter; otherwise, completion of seed germination is likely to be delayed into a second spring.

An additional perspective to consider from this work is that of germplasm preservation. Increasingly, gene banks play a vital role in safeguarding viburnum germplasm in light of threats posed by the VLB and a range of anthropic activities. Internationally, 36% of viburnum species are listed as Vulnerable, Endangered, or Critically Endangered by the Red List [51]. In the U.S., one species is considered Vulnerable (*V. molle*) and another Critically Imperiled (*V. bracteatum*) by NatureServe [35]. For *Viburnum lantanoides*, germplasm preservation may be particularly important for southern populations, where the majority of the taxon’s genetic diversity has been found [24]. NatureServe [35] lists the species as Imperiled (S2) in Ohio, and Critically Imperiled (S1) in Kentucky and New Jersey.

Although viburnum seeds are tolerant to desiccation and cold storage, viability testing and regeneration of preserved seeds must also occur on a regular basis [52]. Tetrazolium staining, as a proxy for viability, is the only official seed testing recommendation for *Viburnum*, though the destructive nature of this test renders it lethal to seeds [16]. Alternatively, germination testing provides additional opportunities to monitor vigor and recover living germplasm. The positive effect of gibberellins including GA3 on seed dormancy alleviation is well documented [9] and its use is commonplace to speed up germination and viability tests, particularly for recovery of low viability seed lots [53,54]. Further research is needed to formally characterize the effect of GA on seed germination in *V. lantanoides*. In particular, can GA be applied to *V. lantanoides* seeds with emerged radicles to further reduce regeneration times? Fedec and Knowles [55] demonstrated that GA3 applied by hand to the hypocotyl base of *Viburnum trilobum* seedlings could successfully substitute for CS in breaking shoot dormancy. Application of gibberellins could be a complementary approach to overcome epicotyl dormancy in *V. lantanoides* when lacking suitable infrastructure to refrigerate seedlings with emerged radicles.

This exploratory study facilitates a conceptual and practical framework for seed-based regeneration of *V. lantanoides* in support of conservation and horticultural efforts. *V. lantanoides* is an underutilized species with many potential applications for restoration and ornamental plantings. Our results further contribute to the collective understanding of the evolution of dormancy in *Viburnum.* The proposed experimental approach offers opportunities for future investigations of interspecific variation, with the ultimate goal of enhancing understanding of specific mechanisms of seed dormancy. Germplasm preservation and conservation initiatives are likely to benefit from future seed studies targeting in situ regeneration, biochemical analyses, and intraspecific variation in *V. lantanoides*.

## 4. Materials and Methods

### 4.1. Germplasm Acquisition and Processing

Fruits were collected from a wild population of *V. lantanoides* in Sullivan County, New Hampshire (~43.5150, −72.1107) during the first week of September in 2021, and again in 2024. At the time of fruit collection, the mature drupes had softened and changed in color to red and purple–black. The same cohort of 10–12 healthy, mature, maternal plants was sampled on both occasions. A subset of each sample was provided to the USDA Woody Landscape Plant Germplasm Repository for public research access, with 2021 and 2024 seeds accessioned as WLP 1447 and WLP 3480, respectively [56].

**Figure 8:**
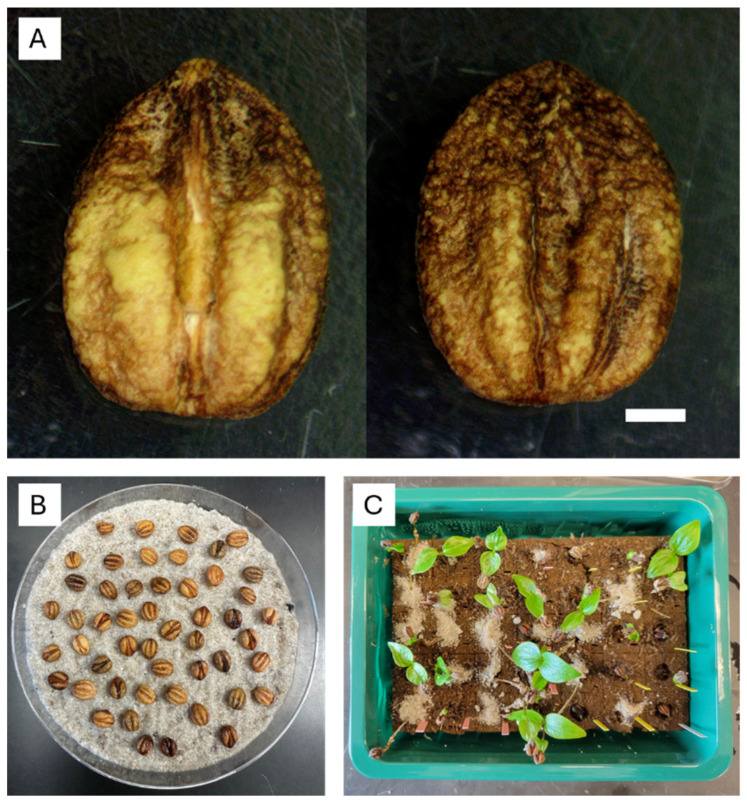
Experimental materials: (**A**) front and back of a cleaned seed (endocarp present), (**B**) Petri dish containing a cohort of 50 seeds, (**C**) seedlings during the cotyledon emergence stage. Scale bar = 1 mm.

Fruits were cleaned by gently macerating drupes in a plastic bag to release exocarp and mesocarp tissue from the endocarps. We use the term ‘seed’ hereafter for convenience, to include the true seed plus endocarp. Seeds were rinsed on a metal screen under running water until completely free of fruit tissue (Figure 8A). Fresh, cleaned seeds were air-dried for 48 h at ambient laboratory conditions, approximately 20–23 °C and 30–35% relative humidity, prior to initiating all experiments.

### 4.2. Seed Permeability

A random subset of 10 seeds collected in 2021 were used for testing seed permeability. Seeds were weighed and added to individual wells in a ceramic well-plate. A small amount of distilled water was added to partially cover each seed. Seeds were individually weighed every 10 min for the first 30 min, every 20 min for the next 1 h, every 1 h for the next 3 h, and then at intervals corresponding to 6, 8, 24, 28, and 32 h from the start of imbibition. At each time point, seeds were blotted dry with a paper towel to remove surface moisture, reweighed, and placed back in their respective wells. Imbibition was expressed for each seed as the percent increase in mass relative to the starting weight.

### 4.3. Seed Dormancy Characterization

Data were collected on four growth chambers, each with 12 h day/night alternating temperatures of 5/2 °C, 15/6 °C, 20/10 °C, or 25/15 °C, respectively. Each growth chamber also provided cool, white, fluorescent lights (20 µmol m^−2^ s^−1^, 400–700 nm) that came on 1 h before the day temperature and remained on until 1 h after the start of the night temperature, for a total photoperiod of 14 h.

Unless otherwise noted, seeds were imbibed for 24 h in distilled water on an orbital stirrer prior to initiating tests for seed dormancy characterization.

Seeds collected in 2021 were used to conduct a qualitative characterization of seed dormancy. Seeds were initiated on a total of eight germination treatments. Treatments MA-W and MA-S were designed to mimic move along (MA-) treatments akin to those in Baskin and Baskin [32], whereby seeds were cycled between incubators to simulate seasonal changes. Specifically, treatment MA-W was incubated at temperatures simulating a winter start (5/2 °C for twelve weeks), → early spring (15/6 °C for four weeks), → late spring (20/10 °C for four weeks), → summer (25/15 °C for twelve weeks), → early autumn (20/10 °C for four weeks), → late autumn (15/6 °C for four weeks) and finally, → winter (5/2 °C for twelve weeks), for a total of 52 weeks. Meanwhile, treatment MA-S simulated a summer start (25/15 °C for twelve weeks), → early autumn (20/10 °C for four weeks), → late autumn (15/6 °C for four weeks), → winter (5/2 °C for twelve weeks), → early spring (15/6 °C for four weeks), → late spring (20/10 °C for four weeks), and finally, → summer (25/15 °C for twelve weeks), for a total of 52 weeks. Control treatments maintained constant temperatures at 5/2 °C (C5), 15/6 °C (C15), 20/10 °C (C20), or 25/15 °C (C25) for 52 weeks. Finally, treatments GA3-1k and GA3-5k expanded treatment C25 to replace seed imbibition in distilled water with 24 h imbibition in solutions of gibberellic acid (GA3) at concentrations of 1000 and 5000 ppm GA3, respectively.

Treatments were randomly assigned to cohorts of 50 seeds, each cohort arranged in a 90 × 15 mm lidded Petri dish containing approximately 5 mm of fine quartz sand moistened with distilled water (Figure 8B). Each treatment was assigned to a total of three Petri dishes. For each Petri dish, seed germination was recorded every seven days for 52 weeks. Seeds were scored as germinated when the length of the protruding radicle was at least 2 mm. Seeds that scored as successfully germinated were removed from the Petri dishes.

For each treatment, the first 24 germinated seeds were transferred to a single 12 × 17 cm plastic germination box with a clear plastic lid. Each box contained 2.5 cm of finely milled sphagnum peat as a growing substrate. Seeds were added in an evenly spaced 4 × 6 array by gently burying the radicle such that the endocarp remained on the substrate surface. Each box accompanied the respective treatment within the growth chambers until the end of the study, at which time the number of seedlings that had developed a shoot, died, or remained alive without shoot development was visually assessed and recorded.

### 4.4. Embryo Development Assessment

For the temperature cycling regimes in treatments MA-W and MA-S, we also conducted a qualitative assessment of embryo development using three additional seed cohorts set up in Petri dishes, as described earlier. At four-week intervals, ten seeds were randomly sampled from each treatment and subjected to destructive measurements. Specifically, seeds were cut in half longitudinally to bisect the embryo. Measurements were collected with a dissecting microscope equipped with a micrometer at 7–10× magnification. We recorded the total length of the embryo, defined as the distance from the tip of the radicle to the top of the cotyledon, and also the length of the true seed, defined as the length of the endosperm collinear to the embryo (Figure 3). The ratio of these measurements was used to calculate the embryo:seed ratio (E:S).

### 4.5. Optimization of Seedling Regeneration Protocols

We used qualitative results derived from the seed dormancy characterization study (2021 data) to inform treatment designs to test seedling regeneration protocols. Specifically, treatments consisted of factorial combinations of warm stratification (WS) applied for 12 or 18 weeks, followed by cold stratification (CS) treatments (5/2 °C) applied for 4, 8, 12, or 16 weeks. WS treatments were applied in two stages; the first stage consisted of either 6 or 12 weeks at summer temperatures (25/15 °C), followed by a second stage of 6-week duration at early-autumn temperatures (20/10 °C).

Seeds collected in 2024 were used in this study. Cohorts of 50 seeds were arranged in Petri dishes, as previously described, and randomly assigned to treatment combinations, such that each treatment combination would be represented by two Petri dishes.

Each Petri dish was assigned a companion 12 × 17 cm plastic germination box into which a subset of 35 seedlings was transferred upon radicle emergence. Germination boxes were similar to those described earlier and consisted of 1.9 × 2.2 × 3.8 cm OASIS Horticubes^®^ (A.M. Leonard, Piqua, OH, USA) (Figure 8C). Seeds were scored as germinated when emergent radicles were at least 2 mm, at which time one seedling was sown per Horticube, until all thirty-five cubes were filled. Radicles were individually suspended within the small hole in a foam cube, and the holes were filled with white quartz sand to stabilize the developing seedlings.

After completion of cold-stratification treatments, all Petri dishes and germination boxes were subjected to a temperature regime consisting of early spring (15/6 °C for four weeks), followed by late spring (20/10 °C for six weeks) and finally, summer (25/15 °C for six weeks). Seeds and seedlings were monitored and scored every seven days for the duration of the experiment. For seedlings, we scored cotyledon emergence when the seed leaves opened and shed their endocarps. *Viburnum* has opposite-leaf arrangements such that the first true leaves emerge as a pair. Emergence of the first (pair of) leaves was scored when at least one of the leaves was at least 1 cm in length.

### 4.6. Statistical Analysis

Data obtained from seeds collected in 2021 were intended for the characterization of seed dormancy and the subsequent assessment of embryo development (Objective 1). These data are presented using descriptive statistics to reflect the qualitative nature of the objective. Inferential statistics were not considered appropriate due to design considerations.

Data obtained from seeds collected in 2024 were intended for optimization of seedling regeneration protocols (Objective 2) and were thus subjected to model fitting and used for inference. Specifically, the cumulative occurrence of each phenological event of interest, namely the emergence of radicle, cotyledon, and true leaves, was recorded on each Petri dish and corresponding germination box on a weekly basis for the duration of the study. In each case, the probability of emergence was estimated using a generalized linear model [57] that assumed a binomial distribution and implemented a canonical logit link function. The linear predictor included the fixed effects of duration of warm stratification (12 vs. 18 weeks). In addition, for cotyledon and true leaf emergence, the linear predictor also included the fixed effect of duration of cold stratification (4, 8, 12 vs. 16 weeks) and the two-way interaction. The method of estimation was maximum likelihood, with the addition of Firth’s method of bias correction in the case of cotyledon emergence to accommodate quasi-complete separation of datapoints. There was no evidence for overdispersion in any of the models fitted. We report the estimated probability of emergence of each phenological stage (and corresponding 95% confidence intervals).

Time-to-event responses, specifically time to emergence of each phenological stage of interest, namely radicle, cotyledon, and true leaves, were expressed in weekly increments. The non-parametric Kaplan–Meier estimator was used to obtain estimates of the cumulative probability of emergence for each phenological stage and corresponding 95% confidence intervals, accounting for censoring of datapoints, where appropriate. A Cox regression model with a shared frailty was fitted to each time-to-event response for testing purposes. Fixed effects were specified as described earlier. The linear predictor also included a shared frailty term to recognize subsampling in the experimental design, thereby differentiating seed (or seedling) as the unit of observation from the clusters defined by Petri dish (or germination box), which in turn served as the experimental units for both warm and cold stratification treatments. The log-frailty random variable was specified to have a log-normal distribution with mean zero. Estimation of variance components was conducted using residual maximum likelihood. Ties were handled using an exact estimation that considered all possibilities of ordering within a tie. Estimated hazard ratios between treatments (and corresponding 95% confidence intervals) are reported to quantify treatment effects of interest. Testing was based on Chi-square statistics estimated within the fitted models, thus accounting for within-cluster correlations in the data.

Treatment comparisons were adjusted using Tukey–Kramer (marginal effects) or Bonferroni’s (simple effects) procedures to avoid inflation of the Type I error rate due to multiple comparisons, as appropriate in each case. Model fitting was implemented using the LOGISTIC, GLIMMIX and PHREG procedures of SAS (Version 9.4, SAS Institute), as appropriate for each data type.

## Data Availability

The raw data supporting the conclusions of this article will be made available by the authors on request.

## References

[1] Dumroese R.K., Landis T.D. (2016). The Native Plant Propagation Protocol Database: 16 years of sharing information. Nativ. Plants J..

[2] Dirr M.A., Heuser C.W. (1987). The Reference Manual of Woody Plant Propagation: From Seed to Tissue Culture.

[3] Xie W.-J., Leus L., Wang J.-H., Van Laere K. (2017). Fertility barriers in interspecific crosses within *Viburnum*. Euphytica.

[4] Center for Plant Conservation (2019). CPC Best Plant Conservation Practices to Support Species Survival in the Wild.

[5] Finch-Savage W.E., Leubner-Metzger G. (2006). Seed dormancy and the control of germination. New Phytol..

[6] Lamont B.B., Pausas J.G. (2023). Seed dormancy revisited: Dormancy-release pathways and environmental interactions. Funct. Ecol..

[7] Willis C.G., Baskin C.C., Baskin J.M., Auld J.R., Venable D.L., Cavender-Bares J., Donohue K., Rubio de Cases R. (2014). The evolution of seed dormancy: Environmental cues, evolutionary hubs, and diversification of the seed plants. New Phytol..

[8] Baskin J.M., Baskin C.C. (2021). The great diversity in kinds of seed dormancy: A revision of the Nikolaeva-Baskin classification system for primary seed dormancy. Seed Sci. Res..

[9] Baskin C.C., Baskin J.M. (2014). Seeds: Ecology, Biogeography, and Evolution of Dormancy and Germination.

[10] Baskin C.C., Baskin J.M. (2022). Plant Regeneration from Seeds: A Global Warming Perspective.

[11] Barton K.E., Macinnis-Ng C., Ostertag R., Kagawa-Viviani A. (2026). Physiological traits for climate-ready restoration. Ecol. Evol..

[12] Silveira F.A.O., Carta A., Dayrell R.L.C., Fernandez-Pascual E., Garbowski M., Hornick T., Klimesova J., Korell L., Ladouceur E., Larson J. (2026). Open letter: A call to integrate plant regeneration into sustainability science and policy. Plants People Planet.

[13] Hamm T.P., Nowicki M., Boggess S.L., Ranney T.G., Trigiano R.N. (2023). A set of SSR markers to characterize genetic diversity in all *Viburnum* species. Sci. Rep..

[14] Egolf D.R. (1956). Cytological and Interspecific Hybridization Studies in the Genus *Viburnum*. Ph.D. Thesis.

[15] Barton L.E. (1933). Experiments at Boyce Thompson Institute on germination and dormancy in seeds. Sci. Hortic..

[16] Bonner F.T., Karrfalt R.P. (2008). The Woody Plant Seed Manual.

[17] Jaganathan G.K., Phartyal S.S., Gehan Jayasuriya K.M.G. (2025). From oversight to insight: Integrating epicotyl emergence to redefine germination and enhance the seed dormancy framework. Seed Sci. Res..

[18] Chien C.-T., Chen S.-Y., Tsai C.-C., Baskin J.M., Baskin C.C., Kuo-Huang L.-L. (2011). Deep simple epicotyl morphophysiological dormancy in seeds of two *Viburnum* species, with special reference to shoot growth and development inside the seed. Ann. Bot..

[19] Santiago A., Ferrandis P., Herranz J.M. (2015). Non-deep simple morphophysiological dormancy in seeds of *Viburnum lantana* (Caprifoliaceae), a new dormancy level in the genus *Viburnum*. Seed Sci. Res..

[20] Chen S.-Y., Liu C.-P., Baskin C.C., Chien C.-T. (2021). Deep complex morphophysiological dormancy in seeds of *Viburnum plicatum* var. *formosanum* (Adoxaceae) from subtropical mountains. Seed Sci. Res..

[21] Seed Information Database. https://ser-sid.org/.

[22] Moskalets T., Moskalets V., Marchenko A., Pelekhatyi V., Yakovenko R. (2023). Harmfulness of the viburnum leaf beetle (Pyrrhalta viburni Payk.) on plants of the *Viburnum* L. genus and elements of its control technology for strategiesin breeding work in the system of fruit and decorative gardening. Sci. Horiz..

[23] Weston P.A., Desurmont G., Hoebeke E.R. (2007). *Viburnum* leaf beetle (COLEOPTERA: Chrysomelidae): Biology, invasion history in North America, and management options. Am. Entomol..

[24] Park B., Donoghue M.J. (2019). Phylogeography of a widespread eastern North American shrub, *Viburnum lantanoides*. Am. J. Bot..

[25] Park B., Sinnott-Armstrong M., Schlutius C., Penagos Zuluaga J.-C., Spriggs E.L., Simpson R.G., Benavides E., Landis M.J., Sweeney P.W., Eaton D.A.R. (2019). Sterile marginal flowers increase visitation and fruit set in the hobblebush (*Viburnum lantanoides*, Adoxaceae) at multiple spatial scales. Ann. Bot..

[26] Sargent C.S. (1889). *Viburnum* *lantanoides*. Gard. For..

[27] Dirr M.A. (2009). Manual of Woody Landscape Plants.

[28] Aiello A.S., Dosmann M.S. (2019). Déjà vu *Viburnums*: A world away but close to home. Arnoldia.

[29] Giersbach J. (1937). Germination and seedling production of species of *Viburnum*. Contrib. Boyce Thompson Inst..

[30] Landis M.L., Eaton D.A.R., Clement W.L., Park B., Spriggs E.L., Sweeney P.W., Edwards E.J., Donoghue M.J. (2021). Joint phylogenetic estimation of geographic movements and biome shifts during the global diversification of *Viburnum*. Syst. Biol..

[31] Gill J.D., Pogge F.L., Schopmeyer C.S. (1974). *Viburnum* L., viburnum. Seeds of Woody Plants in the United States.

[32] Baskin C.C., Baskin J.M. (2003). When breaking seed dormancy is a problem, try a move along experiment. Nativ. Plants J..

[33] Baskin J.M., Baskin C.C., Li X. (2000). Taxonomy, anatomy and evolution of physical dormancy in seeds. Plant Species Biol..

[34] Karlsson L.M., Hidayati S.N., Walck J.L., Milberg P. (2005). Complex combination of seed dormancy and seedling development determine emergence of *Viburnum tinus* (Caprifoliaceae). Ann. Bot..

[35] NatureServe. https://explorer.natureserve.org/.

[36] Rosbakh S., Carta A., Fernandez-Pascual E., Phartyal S.S., Dayrell R.L.C., Mattana E., Saatkamp A., Vandelook F., Baskin J., Baskin C. (2023). Global seed dormancy patterns are driven by macroclimate but not fire regime. New Phytol..

[37] Baskin C.C., Chien C.-T., Chen S.-Y., Baskin J.M. (2008). Germination of *Viburnum odoratissimum* seeds: A new level of morphophysiological dormancy. Seed Sci. Res..

[38] Hidayati S.N., Baskin J.M., Baskin C.C. (2005). Epicotyl dormancy in *Viburnum acerifolium* (Caprifoliaceae). Am. Midl. Nat..

[39] Bolmgren K., Eriksson O. (2015). Are mismatches the norm? Timing of flowering, fruiting, dispersal and germination and their fitness effects in Frangula alnus (Rhamnaceae). Oikos.

[40] Penfield S. (2017). Seed dormancy and germination. Curr. Biol..

[41] Copete M.A., Herranz J.M., Herranz R., Copete E., Ferrandis P. (2021). Effects of desiccation of seeds in nine species with morphophysiological dormancy on germination and embryo growth. J. Plant Ecol..

[42] Jarvis-Lowry B., Harrington K.C., Ghanizadeh H., Robertson A.W. (2024). Viability and dormancy of the Clematis vitalba aerial seed bank. Plant Biol..

[43] Zhao X., Wang F., Wang L., Wang Q., Liu A., Li Y. (2024). Deep simple epicotyl morphophysiological dormancy in seeds of endemic Chinese Helleborus thibetanus. Agriculture.

[44] Copete E., Herranz J.M., Ferrandis P., Baskin C.C., Baskin J.M. (2011). Physiology, morphology and phenology of seed dormancy break and germination in the endemic Iberian species *Narcissus hispanicus* (Amaryllidaceae). Ann. Bot..

[45] Miller F., Rigsby C. (2024). Host plant resistance of *Viburnum* taxa for the viburnum leaf beetle (Coleoptera: Chrysomelidae). Gt. Lakes Entomol..

[46] *Viburnum* Leaf Beetle Susceptibility to Infestation. http://www.hort.cornell.edu/vlb/suscept.html.

[47] Dirr M.A. (2013). Viburnums: Flowering Shrubs for Every Season.

[48] 2024 Census of Horticultural Specialties, Volume 3: Special Studies, Part 3. https://www.nass.usda.gov/Publications/AgCensus/2022/Online_Resources/Census_of_Horticulture_Specialties/index.php.

[49] Brzuszek R.F., Harkess R.L. (2009). Green industry survey of native plant marketing in the southeastern United States. HortTechnology.

[50] Fertakos M.E., Birch S., Dearstyne T., Fehr T., Allen J.M., Bradley B.A. (2026). Current nursery offerings highlight the potential to expand native plant sales. Front. Ecol. Environ..

[51] IUCN Red List of Threatened Species. https://www.iucnredlist.org.

[52] Justice O.L., Bass L.N. (1978). Principles and Practices of Seed Storage.

[53] Rogers D.R., Nonnecke G.R., Goggi A.S. (2025). Accessible seed germination and storage techniques for ex situ conservation of yellow necklacepod (Sophora tomentosa). Hortscience.

[54] Seiler G.J. (2022). Germination and viability of wild sunflower species seeds stored at room temperature and low humidity for 38 years. Seed Sci. Technol..

[55] Fedec P., Knowles R.H. (1973). Afterripening and germination of seeds of American highbush cranberry (*Viburnum trilobum*). Can. J. Bot..

[56] GRIN-Global. https://npgsweb.ars-grin.gov/gringlobal/search.

[57] Stroup W.W., Ptukhina M., Garai J. (2024). Generalized Linear Mixed Models: Modern Concepts, Methods and Applications.

